# Ventilatory efficiency in post‐COVID‐19 athletes

**DOI:** 10.14814/phy2.15795

**Published:** 2023-09-21

**Authors:** Klara Komici, Leonardo Bencivenga, Giuseppe Rengo, Andrea Bianco, Germano Guerra

**Affiliations:** ^1^ Department of Medicine and Health Sciences University of Molise Campobasso Italy; ^2^ Exercise and Sports Medicine Unit Antonio Cardarelli Hospital Campobasso Italy; ^3^ Department of Translational Medical Sciences University of Naples “Federico II” Naples Italy; ^4^ Istituti Clinici Scientifici Maugeri IRCCS‐Scientific Institute of Telese Terme Telese Terme Italy; ^5^ Department of Translational Medical Sciences University of Campania “L. Vanvitelli” Naples Italy

**Keywords:** athletes, COVID‐19, CPET, exercise, VE/VCO_2_ slope, ventilatory efficiency

## Abstract

Limitation in exercise capacity has not been described in athletes affected by SARS‐CoV‐2 infection. However, patients who have recovered from COVID‐19 without cardiopulmonary impairment show exaggerated ventilatory response during exercise. Therefore, we aimed to evaluate the ventilatory efficiency (VEf) in competitive athletes recovered from COVID‐19 and to characterize the ventilation versus carbon dioxide relationship (VE/VCO_2_) slope in this population. Thirty‐seven competitive athletes with COVID‐19 were recruited for this study. All participants underwent spirometry, echocardiography, and cardiopulmonary exercise testing (CPET). *z*‐FVC values and end‐title pressure of CO_2_ (P_ET_CO_2_) were lower in the third tertile compared with the first tertile: −0.753 ± 0.473 vs. 0.037 ± 0.911, *p* = 0.05; 42.2 ± 2.7 vs. 37.1 ± 2.5 mmHg, *p* < 0.01. VE/VCO_2_ slope was significantly correlated to maximal VCO_2_/VE and maximal VO_2_/VE: coefficient = −0.5 *R* ^2^ = 0.58, *p* < 0.0001 and coefficient = −0.3 *R* ^2^ = 0.16, *p* = 0.008. Competitive athletes affected by SARS‐CoV‐2 infection, without cardio‐respiratory disease sequel, may present ventilatory inefficiency (ViE), without exercise capacity limitation. FVC is higher in athletes with better ventilatory performance during exercise, and increased VE/VCO_2_ slope is inversely correlated to max VCO_2_/VE and max VO_2_/VE.

## 1 INTRODUCTION

The coronavirus disease‐2019 (COVID‐19), caused by severe acute respiratory syndrome coronavirus 2 (SARS‐CoV‐2) infection, has determined more than 400 million confirmed cases globally (https://www.who.int/emergencies/diseases/novel‐coronavirus‐2019/situation‐reports). COVID‐19 acute manifestation mainly involves the respiratory system. Neurologic, cardiovascular, hematologic, and gastrointestinal alterations have also been reported (Carfi et al., 2020).

Reduced physical capacity and exercise hyperventilation have been described as common manifestations in post‐COVID‐19 patients (Baratto et al., 2021; Skjorten et al., 2021). Muscle deconditioning represents a possible explanation for the reduction of exercise capacity in COVID‐19 survivors, however, no relevant sequelae on gas exchange and ventilatory response to exercise was found in a previous study (Rinaldo et al., 2021). Among athletes affected by COVID‐19 disease, a significant limitation in exercise capacity has not been described (Gervasi et al., 2021; Komici et al., 2021) and SARS‐CoV‐2 infection is associated with a low risk of cardiovascular consequences (Moulson et al., 2021). However, a recent study revealed that patients who recovered from COVID‐19 without cardiopulmonary impairment present an exaggerated ventilatory response during exercise and impairment of systemic oxygen extraction (Singh et al., 2022).

Ventilatory efficiency (VEf) is defined as the amount of ventilation (VE) required to eliminate a liter of carbon dioxide production (VCO_2_) (Wasserman et al., 2012). The slope of VE to VCO_2_ (VE/VCO_2_) slope has been proposed as the best index for evaluating VEf during incremental exercise (Mezzani et al., 2017; Wasserman et al., 2012). Ventilatory inefficiency (ViE) is defined as an abnormal ventilatory response to exercise, identified by increased VE/VCO_2_ slope during incremental cardiopulmonary exercise testing (CPET) (Coats, 2005; Sun et al., 2002). VE/VCO_2_ slope < 30 is considered normal and higher values indicate the severity of cardiorespiratory diseases and are associated with poor outcomes (Gong et al., 2022; Tumminello et al., 2007). Increased VE/VCO_2_ slope has been attributed to early exercise‐induced metabolic acidosis, enhanced ventilatory reflex sensitivity due to overactivation of the sympathetic nervous system, and ventilation/perfusion mismatch (Collins et al., 2021; Guazzi et al., 2005; Ponikowski et al., 2001). Compared to untrained individuals, athletes demonstrate a lower VE at a given work rate due to less reliance on anaerobic metabolism (Collins et al., 2021; di Paco et al., 2017).

Other studies have reported that VEf reacts independently of fitness level, which tends to respond similarly in athletes (Salazar‐Martinez et al., 2016, 2018). It should be mentioned that the detection of ventilatory inefficiency (ViE) during exercise in athletes is indicative of underlying respiratory and cardiovascular abnormalities and further clinical investigation is warranted (Collins et al., 2021).

In patients recovering from mild COVID‐19 and without evidence of cardiorespiratory diseases or anemia, the presence of ViE was attributed to enhanced chemoreflex sensitivity rather than a centrally mediated hyperventilation or impairment of cardiorespiratory system (Singh et al., 2022).

The ventilatory response to exercise and the pathophysiological bases of ViE in post‐COVID‐19 athletes remains unknown. Thus, the aim of our study was to evaluate the VEf in competitive athletes recovered from COVID‐19 and to characterize the VE/VCO_2_ slope in this population.

## 2 MATERIALS AND METHODS

### 2.1 Study population

Athletes recovered from COVID‐19 and referred to Exercise and Sports Medicine Unit, “Antonio Cardarelli Hospital,” Campobasso, Italy for clinical evaluation before return to competitions between January 2021 and June 2021 were screened for enrollment. Inclusion criteria were (a) age ≥18 years; (b) positive testing to SARS‐CoV‐2 by RT‐PCR SARS‐CoV‐2 RNA from nasopharynx swab; (c) negative RT‐PCR SARS‐CoV‐2 RNA and end of self‐isolation period, as indicated by current National Government Recommendations; (d) willingness to participate in this study. Participants were consecutively enrolled by our center and athletes who were evaluated only by exercise stress testing were not considered for this study.

The study protocol was approved by the Institutional Review Board of the Department of Medicine and Health Sciences University of Molise Protocol number 2021/07, and the participants gave written consent for anonymous clinical data collection.

Resting systolic and diastolic blood pressure (SBP, DBP), resting electrocardiogram (ECG), and body mass index (BMI) were registered for all patients. Individual records regarding symptoms presentation, their duration in days, and previous medical conditions, such as asthma, allergy, and cardiovascular risk factors, were collected as described elsewhere (Komici et al., 2021).

### 2.2 Spirometry

Spirometry was performed before the exercise test in accordance with recommended standards. Clinical spirometers: Sensormedics Viasys Carefusion Vmax Encore 22 and Omnia Quark Cosmed 2019 were used for the measurements of Forced Expiratory Volume in one second (FEV1), Forced Vital Capacity (FVC), FEV1/FVC ratio, and Forced Expiratory Flow at rates 25–75%. All measurements were also expressed as percentages of predicted values and z‐scores (Stanojevic et al., 2022).

### 2.3 Echocardiography

Philips iU22 ultrasound system with cardiac sector transducer sampling at 1–5 MHz was used to perform standard transthoracic echocardiography and European Society of Cardiology Recommendations for Chamber Quantification (Lang et al., 2015) were considered for all measurements. In details: left ventricular (LV) size end‐diastolic and end‐systolic diameter, volume, wall thickness, LV Ejection Fraction (LVEF), right ventricle size kinetics and function, fractional area of contraction (FAC) were measured. Peak flow velocity during the early diastolic filling phase (E) and during the atrial contraction (A), and the deceleration time (DT) were measured for evaluation of LV diastolic function. For each Doppler‐based measurement, estimates were obtained from at least 3 cardiac cycles and averaged.

### 2.4 CPET

Breath‐by‐breath gas analysis system Omnia 1.6.10 Quark CPET Cosmed 2019 was used for gas exchange variables and ventilation measurements. Before the examination, calibration was performed according to the instructions of the manufacturer.

Participants were fitted and familiarized with a two‐way breathing Hans Rudolph 7450 series V2mask and headgear before stepping on a motorized treadmill Cosmed T150med. A 12‐lead ECG recording system Quark T12X wireless12‐lead ECG was used and arterial blood pressure was measured at the end of each stage of the test. Noninvasive saturation of peripheral oxygen at rest (SpO_2_%), peak effort, and at the end of recovery were assessed.

All patients performed an incremental exercise test beginning with 8 km/h speed with stepwise increases of 1 km/h every minute. Treadmill inclination was increased by 1% every minute, after reaching a speed of 14 km/h. Patients were encouraged to continue the exercise test until a maximal effort achievement as indicated by: a) failure of oxygen uptake or heart rate (HR) to increase with further increase in work rate; b) peak respiratory exchange ratio (RER) ≥ 1.10; c) rating of perceived exertion ≥8 (on the 10‐point Borg scale) (Borg, 1982; Wasserman et al., 2012).

Peak VO_2_ was recorded as the highest averaged value across at least 10 seconds during exercise. The first ventilatory threshold (1st VT) estimated by the V‐slope or respiratory equivalents methods, Ventilation (VE)/Volume of exhaled carbon dioxide (VCO_2_) slope were evaluated as indicated by Wasserman et al. (2012) and Clinical Recommendations for Cardiopulmonary Exercise Testing Data Assessment in Specific Patient Populations (Guazzi et al., 2016; Mezzani, 2017). VCO_2_/VE and VO_2_/VE were calculated as mL/L at maximal effort. Predicted values of VE/VCO_2_ slope and VO_2_/VE were calculated as proposed by Habedank et al. (1998) and Sun et al. (2002, 2012). For instance: the predicted VE/VCO_2_ slope = 0.13 × age + 19.9; the predicted lowest VE/VCO_2_ = 27.94 + 0.108 × age − 0.0376 × height (cm); the predicted VO_2_/VE max = 42.18–0.189 × years +0.0036 × height (cm).

### 2.5 Statistical analysis

Initially, Shapiro–Wilk test was performed to explore the normality of data distribution. Categorical variables are expressed as absolute frequencies and percentagess. Variables with normal distribution were expressed as mean and standard deviation (SD). Median and interquartile range (IQR) was used when the continuous distribution of variables could not be assumed. Based on VE/VCO_2_ slope values, the study population was divided into tertiles. One‐way ANOVA with Bonferroni and Sidak correction, Kruskal–Wallis's test, and multiple chi‐square test were used to compare quantitative and qualitative variables, as appropriate. Linear regression analysis was used to determine the association between variables of interest. In addition, we performed an analysis to compare the values measured by the clinical spirometers that were used in our study. Statistical significance was at p ≤ 0.05. Statistical analyses were performed with STATA SE 16.1 StataCorp LLC software.

## 3 RESULTS

Clinical and instrumental characteristics of the whole study population are reported in Table 1. Thirty‐seven male competitive soccer athletes were included with a median age of 24 years, IQR 22–27. Competitive athletes are individuals who regularly exercise more than 10 h/week and participate in official sports competitions (Solberg et al., 2016). Baseline spirometry and echocardiography data were within normal range, and no pericardial infusion or other echocardiographic signs suggestive of pericarditis and/or myocarditis were detected in our population.

**TABLE 1:** Overall population characteristics.

	All population *N* = 37
Age years, median, IQR	24 (20–27)
Gender male %	100
BMI kg/m^2^, mean SD	23.6 ± 1.2
HR basal bpm, mean SD	61.3 ± 9.8
SBP mmHg, mean SD	117.8 ± 10.9
DBP mmHg, mean SD	73.5 ± 7.9
Medical history	
Asthma, *n* (%)	7 (18.9)
Arrythmia, *n* (%)	4 (10.8)
Hypothyroidsm, *n* (%)	3 (8.1)
Smokers *n* (%)	5 (13.5)
Time since first positive, days median 95% CI	21 (18.8–24.1)
Fever *n* (%)	17 (45.9)
Cough *n* (%)	15 (40.5)
Dyspnea *n* (%)	10 (27.1)
Myalgia *n* (%)	20 (54.1)
Fatigue *n* (%)	25 (67.6)
Symptoms duration days, mean SD	3.8 ± 2.4
Persistent Myalgia *n* (%)	9 (24.3)
Persistent cough *n* (%)	4 (10.8)
Baseline echocardiography	
LVM/BSA kg/m^2^, mean SD	108.1 ± 9.8
RVd cm, mean SD	3.8 ± 0.3
FAC %, mean SD	50.5 ± 6.4
E/A, mean SD	1.5 ± 0.27
Dec time ms, mean SD	175.6 ± 22.9
PAPs mmHg, mean SD	24.2 ± 2.7
EF %, mean SD	63.0 ± 4.2
Spirometry	
FVC L mean SD	5.4 ± 0.8
FVC % pred., median, IQR	97 (92–103)
*z*FVC mean, SD	−0.262 ± 0.798
FEV1 L mean SD	4.48 ± 0.81
FEV1,% pred., mean SD	97.9 ± 10.3
*z*FEV1, mean SD	−0.293 ± 0.844
FEV1/FVC % mean SD	83.7 ± 7.6
FEV1/FVC % pred., mean SD	99.7 ± 8.8
*z*FEV1/FVC%, mean SD	0.060 ± 1.196
CPET	
VO_2_ rest mL/kg/min, mean SD	4.5 ± 1.2
VCO_2_ rest L/min mean SD	0.27 ± 0.07
Peak VO_2_ mL/kg/min median, IQR	48 (45–51)
Peak VO_2_% pred median, IQR	109 (104–116)
Peak VE L/min mean SD	111.8 ± 20.6
VE/VCO_2_ slope, mean SD	27.8 ± 3.2
VE/VCO_2_ slope, mean SD	23.8 ± 0.5
Lowest VE/VCO_2_ slope mean SD	24.3 ± 2.5
Peak RER median, IQR	1.1 (1.05–1.14)
Peak HR bpm, mean SD	171.4 ± 8
Peak Borg scale, median, SD	8.6 ± 0.7
Oxygen pulse, mL/kg/beat mean SD	22.4 ± 3.7
1st VT % VO_2_ peak, mean SD	75.6 ± 5
SpO_2_ rest % mean, SD	98.1 ± 0.8
Peak SpO_2_% mean, SD	95.7 ± 1.1
Breathing reserve % mean, SD	38.3 ± 12.8
P_ET_CO_2_ rest mmHg, mean SD	33.3 ± 2.8
P_ET_CO_2_ peak mmHg, mean SD	39.6 ± 3.2

Abbreviations: 1st VT, first ventilatory threshold; BMI, body mass index; BSA, Body Surface Area; DBP, diastolic blood pressure; DT, mitral deceleration time; EF, Ejection Fraction; F.A.C, fractional area of change; FEV1, Forced Expiratory Volume in one second; FVC, Forced Vital Capacity; HR, heart rate; IQR, interquartile range; LVM, Left Ventricular Mass; PAP, systolic Pulmonary Artery Pressure; P_ET_CO_2_, end‐tidal pressure of Carbon Dioxide; pred., predicted; RER, Respiratory Exchange Ratio; RVd, Right Ventricular diameter; SBP, systolic blood pressure; SD, Standard Deviation; SpO_2_, oxygen saturation; VCO_2_: Carbon dioxide uptake; VE, Ventilation; VO_2_, oxygen uptake.

Regarding medical history, 18.9% (7) referred asthma and 13.5% (5) were smokers. Fatigue, myalgia, fever, cough, and dyspnea were the main symptoms reported during SARS‐CoV‐2 infection and the mean symptoms duration was 3.8 ± 2.4 days.

CPET revealed that the median peak VO_2_% predicted was 109 IQR (104–116), peak heart rate (HR) was 171.4 ± 8 beats per minute (bpm), first ventilatory threshold (VT), expressed as % of peak VO_2_, was 75.6 ± 5%, while mean VE/VCO_2_ slope was 27.8 ± 3.2, and breathing reserve was 38.3 ± 12.8%. VE/VCO_2_ slope tertiles were homogeneous for age, BMI, comorbidities, main clinical presentation of COVID‐19 disease, symptoms duration, time in days of medical evaluation since first positive nasopharyngeal swab, and persistence of any symptoms.

Although a trend to higher frequency of fever and fatigue was detected in the second or third tertiles, this result was not significant. Persistence of myalgia and cough were not associated with ViE. Echocardiography data, related to left ventricle ejection fraction (EF), diastolic function, and right ventricle function, were also similar across VE/VCO_2_ slope tertiles. (Table 2).

**TABLE 2:** Comparison of baseline characteristics in different VE/VCO_2_ slope tertiles.

	Tertile I *n* = 13; 25.5 (20.3–26.5)	Tertile II *n* = 14 28.35 (27.2–29.6)	Tertile III *n* = 10 31.2 (29.7–34.2)	*p*‐value
Age, years median 95% CI	22 (19.9–23.9)	24.5 (22.1–27.4)	24.5 (20–5‐30.1)	0.24
BMI, kg/m^2^ mean SD	23.7 ± 1	23.5 ± 1.3	23.4 ± 1.4	0.78
Asthma *n* (%)	3 (23.1)	1 (7.2)	3 (30)	0.33
Arrythmia *n* (%)	3 (23.1)	1 (7.2)	0 (0)	0.30
Hypothyroidism, *n* (%)	1 (7.7%)	2 (14.3)	0 (0)	0.80
Smokers, *n* (%)	0 (0)	2 (7.2)	3 (30)	0. 13
Time since 1st positive, days median 95% CI	21 (17.3–28.8)	19.3 (16.5–22.1)	22.5 (16.1–28.8)	0.74
Fever *n* (%)	4 (30.7)	10 (71.4)	3 (30)	0.07
Cough *n* (%)	4 (30.7)	6 (42.8)	5 (50)	0.64
Dyspnea *n* (%)	3 (23.1)	4 (28.6)	3 (30)	0.92
Myalgia *n* (%)	5 (38.5)	9 (64.3)	6 (60)	0.42
Fatigue *n* (%)	6 (46.2)	10 (71.2)	9 (69.2)	0.09
Symptoms duration days, mean SD	3.7 ± 1.6	4.2 ± 3.4	3.6 ± 1.7	0.84
Persistent Myalgia *n* (%)	2 (15.3)	4 (28.6)	3 (30)	0.702
Persistent Cough *n* (%)	1 (7.7)	3 (21.4)	0	0.424
HR basal b/m, mean SD	61.6 ± 7.6	61.4 ± 7.6	60.8 ± 13.1	0.98
SBP mmHg, mean SD	116.2 ± 12.6	116.1 ± 8.4	122.5 ± 11.4	0.29
DBP mmHg, mean SD	70.8 ± 7.6	75.7 ± 7.6	74 ± 8.4	0.26
LVM kg/m^2^, mean SD	109.5 ± 7.7	106.5 10.9	108.5 ± 11.2	0.73
RVd cm, mean SD	3.7 ± 0.19	3.7 ± 0.29	3.8 ± 0.3	0.93
FAC %, mean SD	51.2 ± 6.4	50.5 ± 5.2	49.7 ± 8.2	0.85
E/A, mean SD	1.5 ± 0.27	1.5 ± 0.17	1.5 ± 0.4	0.91
Dec time ms, mean SD	170.9 ± 20.2	170.3 ± 27.3	187.9 ± 16.2	0.13
PAP mmHg, mean SD	24.2 ± 3.02	23.2 ± 2.2	25.5 ± 2.6	0.12
EF %, mean SD	63.4 ± 3.8	63.4 ± 4.1	62 ± 5.1	0.67

*Note*: Tertiles are expressed as median, minimal, and maximal values. Statistic analysis is performed with one‐way ANOVA with Bonferroni correction. Statistical significance for *p*‐value ≤0.05.

Abbreviations: BMI, body mass index; BSA, Body Surface Area; DBP, diastolic blood pressure; DT, mitral deceleration time; EF, Ejection Fraction; F.A.C, fractional area of change; HR, heart rate; LVM, Left Ventricular Mass; PAP, systolic Pulmonary Artery Pressure; RVd, Right Ventricular diameter; SBP, systolic blood pressure.

### 3.1 Spirometry parameters

FVC values, expressed as % of predicted and *z*‐score, were significantly lower in the third tertile compared with the first tertile. FEV1 was higher in the first tertile compared with the third, whereas other spirometry parameters were not significantly different across VE/VCO_2_ slope tertiles (Table 3). Linear regression analysis did not show a significant relationship between VE/VCO_2_ slope and FVC mean value: *R* ^2^ = 0.03; *p* = 0.9; FVC % predicted: *R* ^2^ = 0.04; *p* = 0.24, *z*‐FVC: *R* ^2^ = 0.05; *p* = 0.18 and FEV1 mean value: *R* ^2^ = 0.08; *p* = 0.09. Measurements regarding FVC and FEV1 and VE/VCO_2_ slope tertiles were not different despite the spirometer used (Table S1).

**TABLE 3:** Comparison of spirometry parameters in different VE/VCO_2_ slope tertiles.

	Tertile I *N* = 13; 25.5 (20.3–26.5)	Tertile II *N* = 14 28.35 (27.2–29.6)	Tertile III *N* = 10 31.2 (29.7–34.2)	*p*‐value
FVC L, mean SD	5.6 ± 0.8	5.6 ± 0.7	4.8 ± 0.7*	0.038
FVC % pred., median, IQR	98 (98–106)	99.5 (93–105)	93.5 (88–95)*	0.02
*z*FVC, mean SD	0.037 ± 0.911	−0.191 ± 0.750	−0.753 ± 0.473*	0.05
FEV1 L, mean SD	4.6 ± 0.6	4.6 ± 0.5	4.1 ± 0.5*	0.03
FEV1% pred, mean SD	99.8 ± 9.7	99.5 ± 10.7	99.5 ± 10.7	0.15
*z*FEV1, mean, SD	−0.032 ± 0.828	−0.306 ± 0.952	−0.613 ± 0.644	0.26
FEV1/FVC % mean SD	83.9 ± 9.5	82.5 ± 7.2	85.1 ± 6.1	0.72
FEV1/FVC % pred. mean SD	99.2 ± 10.9	99.4 ± 8.7	100.9 ± 6.3	0.88
*z*FEV1/FVC%	0.011 ± 1.44	−0.136 ± 1.21	0.398 ± 0.80	0.563

*Note*: *p ≤ 0.05 first Tertile vs. third Tertile. Tertiles are expressed as median, minimal, and maximal values. Statistical analysis is performed with one‐way ANOVA with Bonferroni or Sidak correction. Statistical significance for *p*‐value ≤0.05.

Abbreviations: FEV1, Forced Expiratory Volume in one second; FVC, Forced Vital Capacity; IQR, interquartile range; pred, predicted; SD, standard deviation.

### 3.2 CPET parameters

Peak VO_2_ mL/kg/min and peak VO_2_, expressed as a percentage of predicted VO_2_, were not significantly different across tertiles. In addition, peak HR, oxygen pulse, peak RER, and first VT, expressed as a percentage of peak VO_2_ registered during the exercise test, did not differ across tertiles (Table 4).

**TABLE 4:** Comparison of CPET parameters in different VE/VCO_2_ slope tertiles.

	Tertile I *n* = 13; 25.5 (20.3–26.5)	Tertile II *n* = 14 28.35 (27.2–29.6)	Tertile III *n* = 10 31.2 (29.7–34.2)	*p*‐value
VO_2_ mL/kg/ min, rest mean SD	4.4 ± 1.2	4.4 ± 0.8	4.7 ± 1.6	0.77
VCO_2_ L/min rest mean SD	0.29 ± 0.1	0.27 ± 0.5	0.26 ± 0.8	0.34
Peak VO_2_ mL/min/kg median, IQR	48.3 (46.3–51.6)	47.8 (46.1–51)	46.5 (40.7–50.2)	0.38
Peak VCO_2_ L/min, mean SD	4.2 ± 0.8	3.8 ± 0.6	3.4 ± 0.6*	0.03
Peak VO_2_% pred, IQR	110 (105–118)	108.5 (105–115)	105.5 (105.5–114)	0.46
Peak VE L/min, mean SD	107.2 ± 18.2	115.5 ± 21.9	112.8 ± 22.5	0.58
VE/VCO_2_ slope, mean SD	24.3 ± 2.2	28.5 ± 0.9*	31.4 ± 1.6^#^	<0.0001
VE/VCO_2_, slope predicted	23.6 ± 0.41	23.8 ± 0.45	24.01 ± 0.71	0.171
Lowest VE/VCO_2_ slope mean SD	22.1 ± 1.6	24.9 ± 1.8*	26.3 ± 1.9*	<0.0001
VE/VCO_2_ slope % predicted, mean SD	93.5 ± 6.51	104.7 ± 7.9*	109.8 ± 8.9*	<0.0001
VCO_2_/VE max, median, IQR	39.3 (37.1–41.5)	32.4 (32.1–34.5)*	30.5 (29.9–32.9)*	0.0002
VO_2_/VE max, mean, SD	35.2 5.1	31 3.5*	29.5 3.5^#^	0.005
VO_2_/VE % predicted, mean SD	79.2 11.9	70.5 8.1	67.5 8.6*	0.02
Peak RER median 95% CI	1.1 (1.06–1.17)	1.1 (1.1–1.07)	1.1 (0.98–1.13)	0.48
Peak HR bpm mean SD	172.4 ± 6.4	169.2 ± 8.5	173 ± 9.3	0.45
Peak Borg scale, mean SD	8.7 ± 0.7	8.6 ± 0.6	8.7 ± 0.8	0.88
Oxygen pulse, mL/min/beat mean SD	23.6 ± 3.6	22.1 ± 3.7	21.2 ± 3.6	0.30
1st VT % VO_2_ peak, mean SD	76.1 ± 3.8	75.6 ± 6.3	74.9 ± 4.9	0.87
SpO_2_ rest %, mean SD	98.2 ± 0.8	98 ± 0.6	98.1 ± 0.9	0.87
Peak SpO_2_%, mean SD	96.8 ± 1.1	95.4 ± 1.2	96 ± 1.2	0.33
Breathing reserve %, mean SD	42.2 ± 8.9	39.7 ± 11.8	31.2 ± 14.2	0.08
PETCO_2_ rest, mmHg, mean SD	34.5 ± 2.3	32.6 ± 2.6	32.9 ± 3.4	0.12
PETCO_2_ peak, mmHg mean SD	42.2 ± 2.7	38.9 ± 2.2	37.1 ± 2.5 *	<0.0001

*Note*: Tertiles are expressed as median, minimal, and maximal values. Statistic analysis is performed with one‐way ANOVA with Bonferroni correction. Statistical significance for *p*‐value ≤0.05. *p < 0.01 vs. tertile first; #*p* < 0.01 vs. tertile first and second.

Abbreviations: 1st VT, first ventilatory threshold; IQR, interquartile range; PETCO_2_, partial pressure of end‐tidal Carbon Dioxide; pred., predicted; RER, Respiratory Exchange Ratio; SD, Standard Deviation; SpO_2_, oxygen saturation; VCO_2_, Carbon dioxide uptake; VE, Ventilation; VO_2_, oxygen uptake.

VE/VCO_2_ slope, expressed as percentage of predicted, and lowest VE/VCO_2_ value during incremental exercise test were significantly higher in the third tertile compared with the first and the second tertile compared with the first. Peak VCO_2_ was significantly lower in the third tertile compared with the first (3.4 ± 0.6 vs. 4.2 ± 0.8, respectively; *p* = 0.03).

No significant differences regarding, peak VE, peak oxygen saturation, and breathing reserve were present across tertiles. Maximal VCO_2_/VE (max VCO_2_/VE) showed significantly lower values in the third and second tertile compared to the first one: (30.5 [29.9–32.9] vs 39.3 [37.1–41.5], *p* < 0.001) and (32.4 [32.1–34.5] vs 39.3 [37.1–41.5], *p* = 0.001). P_ET_CO_2_ was lower in the third tertile compared with the first tertile: 42.2 ± 2.7 vs 37.1 ± 2.5 mmHg *p* < 0.01.

**FIGURE 1:**
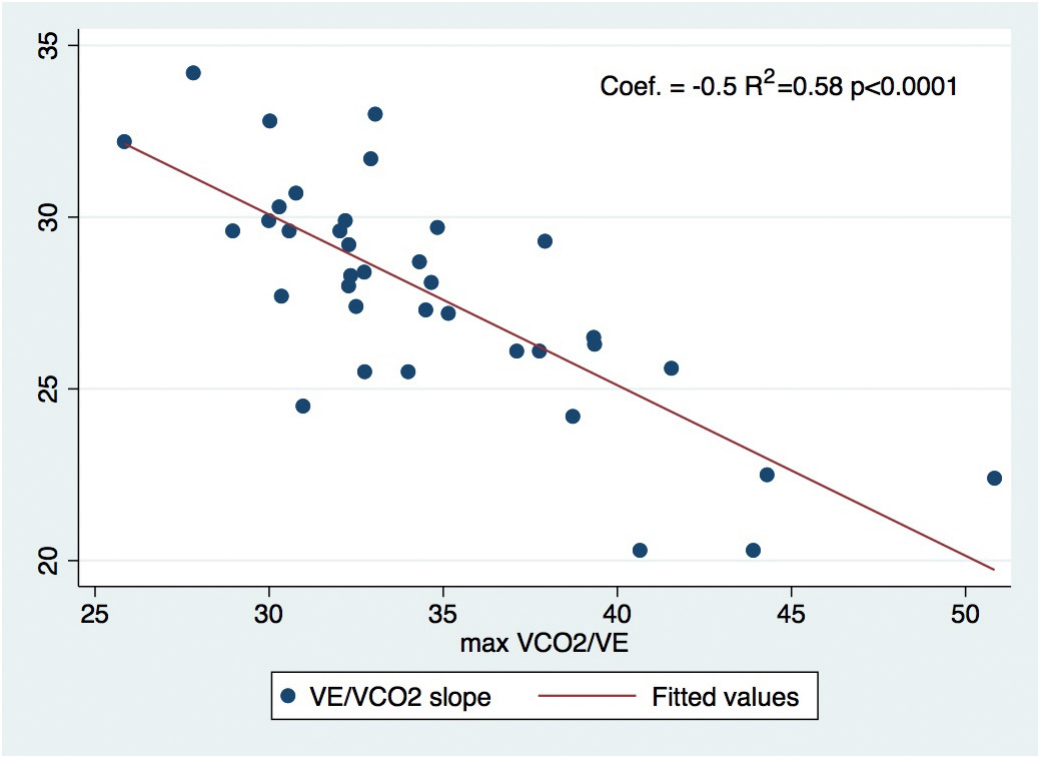
VE/VCO_2_ slope correlation to max VCO_2_/VE. VE/VCO_2_ slope and maximal VCO_2_/VE reached during exercise. VE/VCO_2_ slope is directly and inversely related to maximal VCO_2_/VE.

VO_2_/VE max predicted was significantly lower in the third tertile compared with the first (67.5 ± 8.6 vs. 79.2 ± 11.9 *p* = 0.02). VCO_2_/VE max was significantly related to VE/VCO_2_ slope as indicated by coefficient  = −0.5 *R* ^2^ = 0.58 (*p* < 0.0001) (Figure 1). The correlation between VE/VCO_2_ slope and max VO_2_/VE max was significant (coefficient = −0.3 *R* ^2^ = 0.16 ; *p* = 0.008), whereas peak VO_2_ did not show any significant correlation with VE/VCO_2_ slope (*R* ^2^ = 0.03; *p* = 0.2).

## 4 DISCUSSION

In the current study, we demonstrate that competitive athletes, with post‐SARS‐CoV‐2 infection without cardiorespiratory complications, nearly to recovery may present ViE during incremental exercise without exercise capacity limitation. Athletes with increased VE/VCO_2_ slope are characterized by lower FVC at rest, and VE/VCO_2_ slope is inversely correlated to max VCO_2_/VE and max VO_2_/VE.

VCO_2_/VE and VO_2_/VE termed circulatory equivalents reflect the rate of pulmonary gas exchange at a given ventilation (Hansen et al., 2012). Circulatory equivalents depend on the product of pulmonary blood flow and differences in arteriovenous concentration of O_2_ and CO_2_. Although circulatory equivalents cannot distinguish between deranged pulmonary perfusion or hyperventilation, impairment in VCO_2_/VE and VO_2_/VE relationship reflects abnormality in the adaptation of pulmonary perfusion during exercise (Tan et al., 2018). Lower values of circulatory equivalents are reported among patients with heart failure (Tan et al., 2018), repaired noncyanotic congenital heart disease (Mezzani et al., 2015) and they are able to stratify the severity of heart failure patients (Hansen et al., 2012). However, in the studies conducted by Hansen et al. (2012) and Sun et al. (2012), predicted and reference values of circulatory equivalents were determined in healthy populations including also high fit subjects (Sun et al., 2012) and their measurements may be useful for the noninvasive evaluation of cardiorespiratory function during incremental exercise.

VE/VCO_2_ response to exercise is a relevant physiological parameter. VE increases in proportion to CO_2_ production and O_2_ consumption in order to maintain acid–basic balance and arterial blood gas during exercise (Collins et al., 2021). Ventilatory requirement to remove CO_2_ production is elevated in the presence of hyperventilation or increased dead space (Mezzani, 2017). Initially, hyperventilation is present at the start of exercise, and arterial partial pressure of CO_2_ is reduced. After the stabilization of breathing the increase in VE is appropriate to metabolic demand. At maximal effort, because of excessive metabolic acidosis, VE increases disproportionately to CO_2_ production (Collins et al., 2021).

Increased VE/VCO_2_ slope suggests exercise intolerance and underlying cardiovascular or respiratory diseases. Athletes are characterized by superior cardiovascular fitness and compared to untrained individuals show lower VE during incremental exercise (Collins et al., 2021; di Paco et al., 2017; Martin et al., 1979). At maximal exercise intensities, endurance‐trained athletes may exhibit a blunted VE to excessive metabolic acidosis (Mahler et al., 1982). This alternation has been explained by the improvement of respiratory muscle tolerance to exercise and altered chemoreflex function to hypoxia and hypercapnia related to physical training (Clark et al., 1980).

Studies investigating ViE in post‐COVID‐19 patients have reported conflicting results. In patients with different COVID‐19 severity, measurements of VE/VCO_2_ slope were within normal range and no relevant changes were observed (Rinaldo et al., 2021). Another study reported a significant reduction of peak VO_2_ during exercise in about one third of post‐COVID‐19 patients, without a reduction of breathing reserve (Skjorten et al., 2021). In our study peak VO_2_ reached during incremental exercise, oxygen pulse evaluation, and the detection of first VT showed normal values, and no differences were detected across VE/VCO_2_ slope tertiles. These results indicate a good exercise tolerance and normal stroke volume. Furthermore, the echocardiography examination did not reveal pulmonary hypertension, pericarditis, or myocarditis, spirometry parameters ranged within normal values, and oxygen desaturation was not revealed. From the currently available data, cardiac injury related to COVID‐19 disease is rare among athletes. Limitation of physical performance related to cardiorespiratory complications seems not to occur, at least across athletes with mild to moderate COVID‐19 disease (Komici et al., 2021; Moulson et al., 2021).

Previous studies have described the reduction of FVC in about 11% of patients with severe COVID‐19, while FEV1 was significantly lower in patients with severe to critical COVID‐19 compared to mild or moderate disease (Guler et al., 2021; Wu et al., 2021). However, other studies did not report significant modifications in FEV1 or FVC among post‐COVID‐19 patients (Frija‐Masson et al., 2020; You et al., 2020). A recent meta‐analysis reported that the prevalence of restrictive pattern was 0.15 (95% CI 0.09–0.22), and the obstructive pattern 0.07 (CI 0.04–0.11) [(Torres‐Castro et al., 2021)]. In our population, FVC was lower among athletes with worse VEf compared to those with better ventilatory patterns during exercise, without modifications in peak VE. Cardiorespiratory performance is associated with preservation of lung health (Benck et al., 2017) and a large community‐based cohort of adults found that lower FVC and FEV1 were associated with lower VEf (McNeill et al., 2022). Inflammatory state related to SARS‐CoV‐2 infection may trigger modifications in functioning of respiratory muscles and airway perfusion. Indeed, P_ET_CO_2_ was lower in the group with worse VEf, suggesting a higher work of respiratory muscles and hyperventilation. This ventilatory pattern could be related to modifications of chemoreceptor sensitivity after SARS‐CoV‐2 infection. Indeed, excessive hyperventilation during exercise was reported in post‐COVID‐19 patients with no cardio‐respiratory complications or disease (Singh et al., 2022). VE/VCO_2_ slope was significantly increased in post‐COVID‐19 patients compared to healthy controls and enhanced chemoreflex sensitivity, rather than increased dead space, was suggested as a possible mechanism explaining exercise hyperventilation (Baratto et al., 2021).

Intensive training has been shown to enhance static and dynamic lung volumes (Courteix et al., 1997), and after 8 months of competitive training, FEV1, FVC, and VEf significantly improved in elite athletes (di Paco et al., 2017). In our opinion detraining did not influence the ventilatory parameters in our study since VE/VCO_2_ slope tertiles were homogenous regarding the patient's evaluation and days since the first positive nasopharynx swab, indicating the same period without training. In addition, 6 weeks of detraining did not modify VEf in young soccer players (Alvero‐Cruz et al., 2019). Of interest, our results show a progressive lower max VCO_2_/VE and max VO_2_/VE in groups with worse VEf. Tertiles of VE/VCO_2_ slope were homogenous for the first VT detection, indicating that differences in metabolic acidosis time point during exercise do not influence the ViE. Furthermore, the inverse correlation between max VCO_2_/VE and max VO_2_/VE indicates that probably an early ventilation‐perfusion mismatching mechanism may characterize post‐COVID‐19 patients with ViE. Indeed, endothelial dysfunction has been associated with SARS‐CoV‐19 infection, and pulmonary vessels injury has been described in COVID‐19 patients (Ackermann et al., 2020). In addition, modification of ergoreflex sensitivity may influence impairment of VE and VCO_2_ relationship. Indeed, in chronic heart failure sympathetic nervous system imbalance induced lower chemoceptive CO_2_ setpoint and enhanced ventilatory reflex sensitivity (Witte et al., 2008). Fatigue during acute illness failed to show statistical significance related to worse VEf (*p* = 0.09), however, this result may be influenced by the limited number of subjects in our study population. Indeed, stimulation of skeletal muscle group III–IV afferents, induces ventilation via medullary respiratory centers, and overactivation of these muscle groups may result in excessive ventilatory drive (Rodriguez et al., 2021; Singh et al., 2022). Furthermore, the prolonged physical exertion may influence the immune response in athletes and modification of neuro‐hormonal axis, conditions which in combination with SARS‐CoV‐2 infection may exert a negative impact on VEf (Cannizzaro et al., 2018).

Dysfunctional breathing (DB) is a term describing breathing disorders characterized by dyspnea and other non‐respiratory symptoms in the absence or in excess of respiratory or cardiac disease (Boulding et al., 2016). The diagnosis of DB is challenging and no gold standard diagnostic method exists, however, implementation of CPET has been suggested to improve the diagnosis and the management of DB. Hyperventilation, increased VE/VCO_2_ slope, irregular breathing pattern characterized by highly variable breathing frequency, and tidal volume for a given VE are considered elements of DB evaluated by CPET (Ionescu et al., 2021). Of note dysfunctional breathing pattern has been described in post‐COVID‐19 patients with persisting dyspnea (Frésard et al., 2022). In our study, persisting dyspnea was not present, and BORG score was not significantly different across VE/VCO_2_ tertiles. However, the presence of lower P_ET_CO_2_ in higher VE/VCO_2_ slope tertile suggests an inadequate VE. Anyhow, the relationship between persisting symptoms and physical capacity after COVID‐19 needs further investigation.

It has been suggested that a graded exercise program should be individualized and implemented for individuals participating in high‐level recreational or competitive athletics (Writing Committee et al., 2022). Our findings may help to identify better athletes who need a gradual return to play and adequate training programs for the improvement of respiratory patterns, supported by athletic trainers and sports medicine physicians.

The present study comes with some limitations. First, data are drawn from only male participants. Despite we calculated the predicted VE/VCO_2_ slope which considers age, gender, and height (Sun et al., 2002), our results can be only generalizable to male athletes. The correlation between VE/VCO_2_ slope and maximal VCO_2_/VE is characterized by a modest R2, and this may be influenced by the number of participants. No CPET instrumental examinations were available for the recruited patients before they contracted SARS‐CoV‐2 infection, however, the medical history of all included patients was negative for significant cardio‐respiratory impairment symptoms such as dyspnea, fatigue, and exercise intolerance. Blood gas analysis and plasmatic lactate concentration were not collected in our study. However, P_ET_CO_2_ has demonstrated good reliability as an indirect measure of arterial CO_2_ (Benallal & Busso, 2000). In addition, no structured questionnaires were administrated for the presence of exercise intolerance related to COVID‐19 disease.

### Conclusions

Competitive male athletes affected by SARS‐CoV‐19 infection, without cardio‐respiratory disease sequel, may present ViE, without exercise capacity limitation. FVC is higher in athletes with better ventilatory performance during exercise. Increased VE/VCO_2_ slope is inversely correlated to max VCO_2_/VE and max VO_2_/VE. Future studies on larger population are warranted to define better the ventilatory drive in post‐COVID‐19 athletes and to investigate the pathophysiological basis of these alternations.

## AUTHOR CONTRIBUTIONS

Klara Komici and Germano Guerra conceived this study. Klara Komici, Germano Guerra, and Andrea Bianco performed data acquisition. Klara Komici, Leonardo Bencivenga, and Giuseppe Rengo performed data analysis and interpretation. Klara Komici, Giuseppe Rengo, and Germano Guerra wrote the first draft of the manuscript. All the authors revisited the work critically for important intellectual content. All the authors approved the final version of the manuscript.

## FUNDING INFORMATION

This research received no external funding.

## CONFLICT OF INTEREST STATEMENT

The authors declare no conflict of interest.

## ETHICS STATEMENT

This study was approved by the Institutional Review Board of the Deparment of Medicine and Helath Sciences University of Molise Protocol number 2021/07. All procedures were performed in conformity with Declaration of Helsinki and participants gave written consent for anonymous clinical data collection.

## Supporting information


Table S1.

## Data Availability

The data that support the findings of this study are available from the corresponding author upon reasonable request.
