# Supplementary material for: Ventilatory efficiency in post‐COVID‐19 athletes
Source: Physiol Rep. 2023 Sep 21;11(18):e15795. doi: 10.14814/phy2.15795 (PMC10513909; doi:10.14814/phy2.15795)
Supplement: Supplementary file 1 — Table S1. [file PHY2-11-e15795-s001.docx]

Supplementary Material Table 1. Comparison of measurements obtained by different spirometers.

|  | FVC | FEV1 | FVC/FEV1 |
| --- | --- | --- | --- |
| Spirometr I | 5.43 ±0.87 | 4.5 ± 0.62 | 83.53± 6.5 |
| Spirometr II | 5.37 ±0.78 | 4.47 ±0.50 | 83.87 ± 8.76 |
| p-value | 0.85 | 0.87 | 0.89 |
|  | VE/VCO2 slope Tertile I (n=13) | VE/VCO2 slope Tertile II (n=14 | VE/VCO2 slope Tertile III (n= 10) |
| Spirometr I vs II | 53.8 % | 42.8 % | 60% |
| p-value | 0.69 | | |

Spirometer I: Sensormedics Viasys Carefusion Vmax Encore 22

Spirometer II: Omnia Quark Cosmed 2019
